# Animal Health Surveillance in Scotland in 2030: Using Scenario Planning to Develop Strategies in the Context of “Brexit”

**DOI:** 10.3389/fvets.2017.00201

**Published:** 2017-11-27

**Authors:** Lisa A. Boden, Harriet Auty, Aaron Reeves, Gustaf Rydevik, Paul Bessell, Iain J. McKendrick

**Affiliations:** ^1^School of Veterinary Medicine, College of Medical, Veterinary and Life Sciences, University of Glasgow, Glasgow, United Kingdom; ^2^Epidemiology Research Unit, Scotland’s Rural College (SRUC), Inverness, United Kingdom; ^3^The Roslin Institute, University of Edinburgh, Edinburgh, United Kingdom; ^4^Biomathematics and Statistics Scotland, James Clerk Maxwell Building (JCMB), Edinburgh, United Kingdom

**Keywords:** futures, scenario planning, uncertainty, resilience, public health, notifiable diseases, surveillance, Brexit

## Abstract

Animal health surveillance is necessary to protect human and animal health, rural economies, and the environment from the consequences of large-scale disease outbreaks. In Scotland, since the Kinnaird review in 2011, efforts have been made to engage with stakeholders to ensure that the strategic goals of surveillance are better aligned with the needs of the end-users and other beneficiaries. The aims of this study were to engage with Scottish surveillance stakeholders and multidisciplinary experts to inform the future long-term strategy for animal health surveillance in Scotland. In this paper, we describe the use of scenario planning as an effective tool for the creation and exploration of five plausible long-term futures; we describe prioritization of critical drivers of change (i.e., international trade policy, data-sharing philosophies, and public versus private resourcing of surveillance capacity) that will unpredictably influence the future implementation of animal health surveillance activities. We present 10 participant-developed strategies to support 3 long-term visions to improve future resilience of animal health surveillance and contingency planning for animal and zoonotic disease outbreaks in Scotland. In the absence of any certainty about the nature of post-Brexit trade agreements for agriculture, participants considered the best investments for long-term resilience to include data collection strategies to improve animal health benchmarking, user-benefit strategies to improve digital literacy in farming communities, and investment strategies to increase veterinary and scientific research capacity in rural areas. This is the first scenario planning study to explore stakeholder beliefs and perceptions about important environmental, technological, societal, political, and legal drivers (in addition to epidemiological “risk factors”) and effective strategies to manage future uncertainties for both the Scottish livestock industry and animal health surveillance after Brexit. This insight from stakeholders is important to improve uptake and implementation of animal heath surveillance activities and the future resilience of the livestock industry. The conclusions drawn from this study are applicable not only to Scotland but to other countries and international organizations involved in global animal health surveillance activities.

## Introduction

Animal health surveillance systems are critical at regional, national, and global levels to identify and mitigate biological and chemical hazards (such as animal or zoonotic diseases or syndromes, toxins, or contaminants) to ensure public health and food security and safety. These systems are designed to address societal priorities such as the development of early warning tools for exotic, novel and reemerging diseases, the facilitation of effective disease control, and the monitoring of temporal or spatial disease trends. Surveillance data underpin international trade regulations and are necessary for the development of contingency plans to protect not only human and animal health but also rural economies from the consequences of large-scale disease outbreaks and to mitigate the impacts of animal disease and climate change on each other and the environment.

In Scotland, animal health surveillance for livestock relies primarily on farmers to contribute to passive surveillance by submitting animal materials to eight regional disease surveillance centers (DSCs) for diagnostic and postmortem analysis. This is complemented by abattoir-based recording of diseases significant for human or animal health, including statutory reporting of notifiable diseases, reporting of zoonoses, passive surveillance of wildlife diseases, and active surveillance for specific pathogens or diseases (e.g., *Trichinella spiralis*, Bovine Viral Diarrhea). In addition, industry-led schemes exist to feed disease data back to farmers [for example, pig assurance schemes (1)] but currently these data are rarely integrated with other surveillance systems. Thus, surveillance is carried out through a variety of different systems and implemented by different actors with relatively little integration between systems.

Animal health surveillance in Scotland has been the subject of scrutiny in recent years. Funding for surveillance comes from both Scottish Government and from fees paid by farmers for diagnostic services (2, 3). A comprehensive review in 2011 concluded that the “existing system for delivering veterinary surveillance cannot continue in its present form without significant additional resources, and these (were) very unlikely to be forthcoming in the present financial climate….” The review further concluded that there was “considerable scope to provide disease surveillance more efficiently” (4). Since that time, efforts have been made to engage stakeholders to improve transparency and accountability and to better align strategic goals with the needs of end-users of veterinary surveillance (4).

Given the limited available human and economic resources to support government surveillance frameworks in Scotland (4) and the UK (5), difficult choices must be made about which risks to prioritize in the future. There are numerous established and emerging methods for surveillance data collection, interpretation, and analysis that underpin these decisions (6). These data inform parameters in probabilistic mathematical models and risk analyses so that predictions can be made about the risk of future disease incursions and spread (7). Quantitative approaches have also been used to assess complex sources of economic and epidemiological evidence and evaluate existing surveillance methods (5, 8–10) to underpin disease freedom claims to meet free trade agreements (11). Qualitative (participatory) methods also exist for eliciting and prioritizing expert opinion about future disease risks. These approaches bring together individuals from specialized areas of expertise to consider a single, likely future (12). Both quantitative and qualitative epidemiological approaches are often strongly influenced and constrained by the investigators’ perceptions of the system at risk (13) and reliance on the past as an accurate guide for the future course of events (7, 12).

Scenario planning is a participatory methodology, widely adopted at the science–policy interface in relation to science, technology, and environmental management. It promotes democratic values such as stakeholder-led knowledge generation and analysis [(14–17), at p. 138]. It is a systematic approach that enables participants to anticipate different futures and challenge preconceived assumptions and expectations about the system at risk. Unlike traditional probabilistic approaches, it is best suited for “highly complex, uncertain situations” in which influencing forces and “subjective judgments” cannot be predicted or quantified but are important to incorporate [(18), at p. 818]. Examples of influencing forces include changes in public attitudes toward data privacy and security, governance regimes over information practices and surveillance, and advances in telecommunications technology. These cannot be easily parameterized in existing epidemiological models, yet will affect opportunities for future surveillance data collection and sharing (19, 20). Other unexpected shocks, such as terrorist activities, political upheavals, conflict/war, natural disasters, or extreme weather events, can have unintended and indirect consequences on future disease risks (20). The UK’s decision to leave the European Union (EU) (“Brexit”) is a contemporary example of a “shock” that was largely unexpected because of assumptions about British politics and voting preferences (21). The terms of negotiation for any Brexit deal are still in a state of flux and, to some extent, were not planned for (22). As a result, there is great uncertainty about the scope and magnitude of the societal, economic, environmental, and political implications for farmers in Scotland and the UK making it challenging to consider how to create an animal surveillance system, which will be resilient in the long term.

The aim of this study was to explore the long-term future of animal health surveillance in Scotland and develop robust strategies to mitigate disease challenges and maximize opportunities for the success of future Scottish livestock industries. In this paper, we present a description of foresighting activities and the details of a scenario planning workshop led by EPIC, Scotland’s Centre of Expertise on Animal Disease Outbreaks (www.epicscotland.org), in collaboration with Scottish and UK stakeholders. We describe the five plausible, alternative long-term futures generated in the workshop and propose 10 strategies to improve the long-term resilience of animal health surveillance and contingency planning for animal and zoonotic disease outbreaks in Scotland in future. These strategies are encapsulated by three visions to improve intelligent data collection, investment of resources, and data access and use. We conclude with a discussion of the value of scenario planning, as a mechanism for proactive reflexive risk governance and as a tool for long-term public health contingency planning. This is the first scenario planning study to explore stakeholder beliefs and perceptions about important environmental, technological, societal, political, and legal determinants (in addition to epidemiological “risk factors”), while also providing an opportunity to assess the potential perceived impacts of Brexit. This insight from stakeholders is important to improve uptake and implementation of animal heath surveillance activities and the future resilience of the livestock industry. The conclusions drawn from this study are applicable not only to Scotland but also to other countries and international organizations involved in global animal health surveillance activities.

## Materials and Methods

A scenario planning workshop was held in Edinburgh, Scotland over two consecutive days in October 2016. Scenario planning is a methodology that encourages individuals to think about uncertainties, and “influence current behavior or act in the interests of a better future, or at least improve preparedness for imaginable adverse eventualities” [(17), at 2.1]. The process facilitates the systematic examination of current trends and foreseeable developments to create plausible road-maps to a diverse set of anticipated scenarios. These scenarios are not intended to be predictions of the future, but rather reflect the diversity of possible futures that can be used to think about strategies that could be implemented today to maximize opportunities, or mitigate threats, in the future.

**Figure 1:**
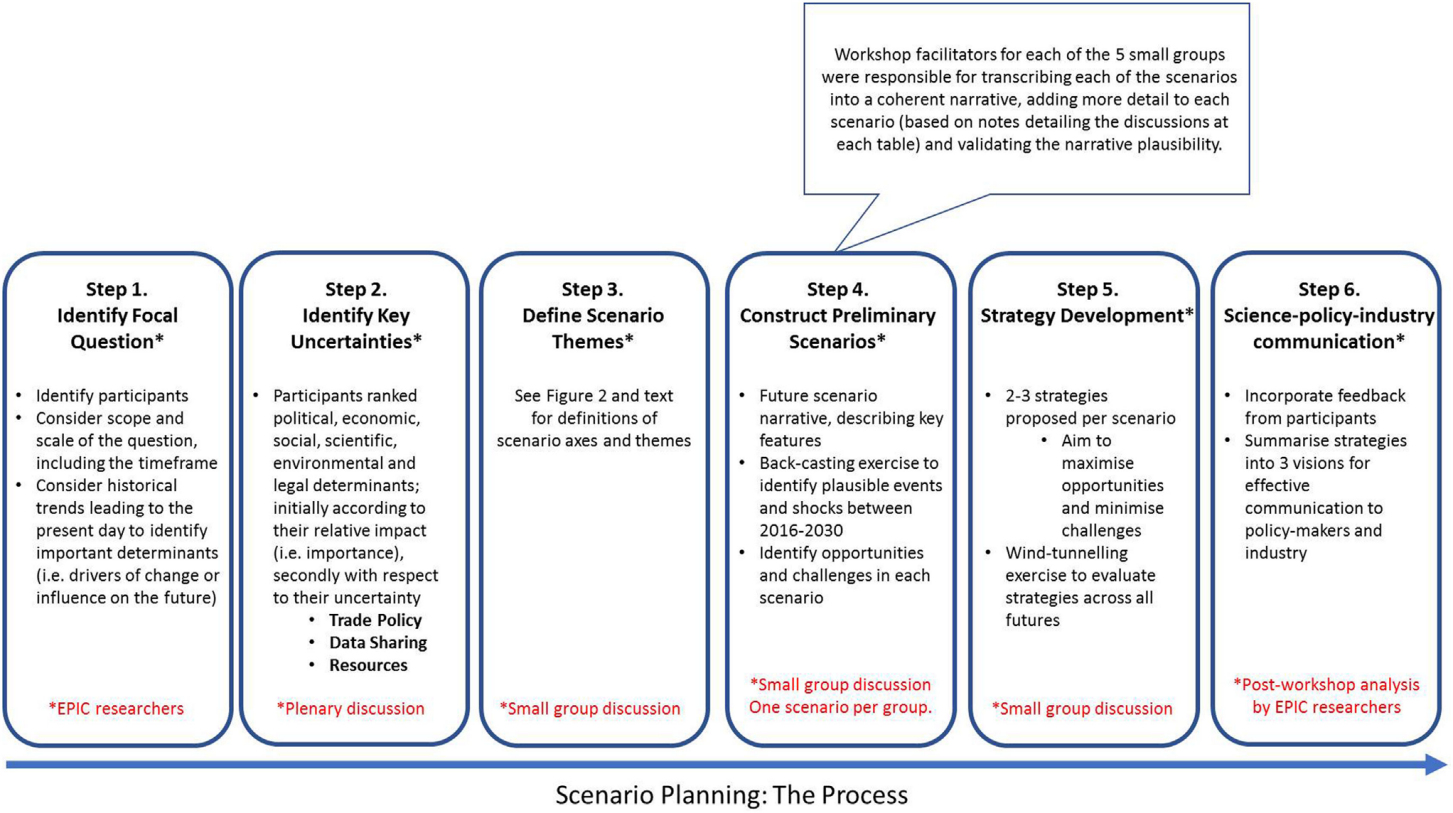
Scenario planning: The process.

There are numerous different methods of conducting scenario planning (23–31). The EPIC workshop included standard and accepted elements of this process as described by Schoemaker (25), Schwartz (32), Foster (33), and Vanston et al. (34). These include “defining the scope of the question, identification of stakeholders, identification of fundamental trends, identification of key uncertainties (political, economic, social, scientific/technological, environmental and legal determinants), construction of initial scenario axes and then themes, development of preliminary (learning) scenario narratives, checking for internal consistency and plausibility of narratives through a back-casting exercise, and use of scenario narratives as decision tools” [(25), as described in (30)]. The choice of these elements is based on a plausibility-based “intuitive logics” approach that enables participants to create narratives that “describe unfolding chains of causation, which resolve themselves into distinct future outcomes” (35). The primary advantage of employing a plausibility-based approach, instead of other qualitative or quantitative methods, is that it enables consideration of multiple challenging futures (35). Figure 1 describes the key features of the scenario planning process undertaken in this study.

### Recruitment

Potential participants (*n* = 50) from Scotland, England, Wales, and Northern Ireland were purposively (non-randomly) selected based on their area of expertise across multiple disciplines and associations with different organizations within the broad research, policy, and industry network available to EPIC, Scottish Government, partner institutes and agencies. A participant from the Netherlands was also invited because the Dutch model (a private company delivering surveillance, and the balance between industry and government stakeholders in driving surveillance activities being different to that in the UK) was considered to be a useful counterpoint to the current UK experience, broadening the range of opinions and scenarios being discussed in the workshop. Participants were selected purposively because of the nature and scope of the question, the limited number of qualified individuals that could contribute to the study, and the need for a heterogeneous group of stakeholders in Scotland. The expertise required in the workshop was based on the scope of the historical drivers (see below) and included anthropology, data protection, economics, engineering, environmental health, ethics, farming, food safety, law and ethics, plant health, policy-making, public health, food retail, social science, technology and innovation industries, veterinary medicine, and wildlife conservation. Of the 50 invited participants, 46 accepted the invitation and attended the workshop.

The project received ethical approval from the University of Glasgow. Within the workshop, all participants agreed to the following condition: “… participants are free to use the information received, but neither the identity nor the affiliation of the speaker(s), nor that of any other participant, may be revealed” (36).

### Scope of the Focal Question

Participants were tasked with engaging in strategic thinking through a series of carefully crafted exercises to explore the focal question “What is the future of animal health surveillance in Scotland in the year 2030?” The year 2030 was selected as giving sufficient time for drivers to influence the future while being sufficiently proximal in time to have elements of familiarity for policy-makers and stakeholders. Animal health surveillance was defined as the continuous detection of the occurrence and distribution of hazards (including diseases, infections or health syndromes) for livestock, wildlife, domestic animals, and human public health (4). The purpose of the focal question was to elicit a dialog about the future strategy for surveillance rather than any discussion of specific operational or tactical elements of surveillance. The sensitivity of surveillance systems for the identification or prioritization of individual hazards, design and implementation of sampling, data collection or analysis to detect exotic, endemic or novel emerging diseases, monitor endemic diseases, and/or demonstrate disease freedom [as described in (37)] is not within the scope of this study. The scope therefore included consideration of the future need for surveillance, how this might be delivered and how the way surveillance is delivered might affect society, public and animal health, and the economy (37).

### Historical Trends and Key Uncertainties

Fundamental trends were investigated through the creation of a visual historical timeline (30, 31). This process involved the identification, discussion, and assessment of important past events and influences (i.e., drivers) on the development of the present day animal disease surveillance strategy. The timeline included directly relevant events, but also exogenous factors which could plausibly have had an indirect influence on surveillance services. This historical timeline was created outwith the workshop, but was subsequently modified based on participant feedback during discussion. The historical timeline was also used to “ground-truth” the list of future drivers. This list was compiled in advance of the workshop and discussed with participants in small groups. A detailed description of each of the drivers is available in the online report and at the EPIC website (see also Table 1 for examples).

**Table 1:** Critical drivers (high impact, high uncertainty drivers), which were clustered to form the three axes used in scenario development.

Axis	International trade policy and the importance of the export market	Sources for, and availability of, resources for disease surveillance, including expertise and infrastructure	Approaches to data sharing
Science/technology			New diagnostic technologies Uptake of precision farming Uptake of smart technology Data sharing between public health and veterinary partners

Society/policy	Brexit Scottish Independence		Data protection regulations Public perception of data sharing Numbers of corporate and superfarms

Economics	Global trade of livestock products and live animals Change in global trading patterns Focus on global food security	Increased global economic prosperity Perception of surveillance as a private or public good Risk-based prioritization of surveillance by government Availability of European Union resources to mitigate for and control animal disease outbreaks Prioritization of national and international resources as a result of human pandemics Expenditure on veterinary education, research and development Farm gate milk prices (and vertical integration of supermarket chain)	

**Figure 2:**
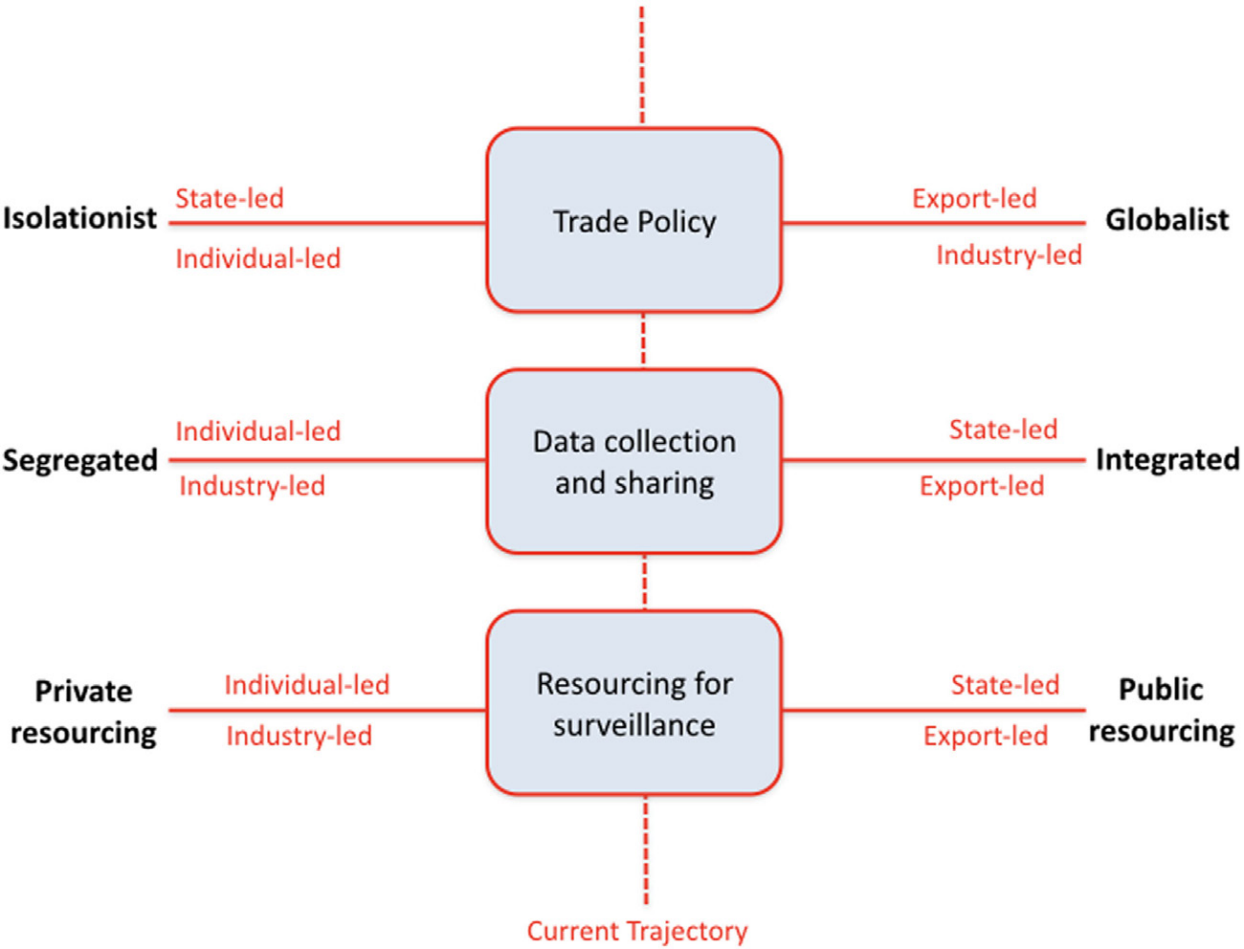
Scenario themes as defined from critical uncertainties (high impact, high uncertainty drivers).

Participants ranked drivers, first according to their relative impact (i.e., importance), and then according to their uncertainty (i.e., the greater the range of plausible outcomes of a driver, the greater the uncertainty). When there was substantially polarized discussion over the uncertainty associated with a driver, the driver was assessed *a priori* as having high uncertainty. High impact, high uncertainty drivers (also known as critical or key uncertainties) were clustered by participants and investigators to create three axes representing a continuum of possibilities between two extreme endpoints. In a participatory exercise, participants were divided into different groups (~8–10 people) to describe future scenario axes (Figure 2). Scenario themes were then defined by creating a combination of different positions on each of the three axes. Scenario development was guided by plausibility, internal consistency, diversity and potential for stimulating discussion about each future (30). High impact, low uncertainty drivers were not eliminated, but were considered in the discussion and development of each scenario. Based on different combinations of realized critical drivers, each small group of workshop participants constructed a scenario to produce five different scenarios in total.

### Preliminary Scenarios

Best- and worst-case scenarios were avoided to ensure that realistic combinations of threats and opportunities were represented. Participants described the key features of one of the five scenarios in a small group exercise. These fundamental scenario characteristics are described in Table 1. Once preliminary scenarios for each future were characterized, a “back-casting” exercise for each scenario was undertaken by the relevant participant group to identify specific hypothetical future events between 2016 and 2030. This back-casting was carried out with the aim of establishing a plausible sequence of events leading from the current situation to the hypothetical future. This exercise serves to add “depth” to the scenarios, challenge and resolve different and potentially conflicting viewpoints about the road to the future and act as a quasi-validation of the outputs. All scenarios considered the future of Scotland and the UK outside of the EU, i.e., post-“Brexit.” All participant views of the future were incorporated in the scenario, provided they were plausible and consistent within the constraints of scenario axes.

Participants added more detail to each scenario during small group discussions throughout the workshop. At the end of the first day, researchers considered each of the draft scenario futures developed by participants and identified areas where further consideration of plausibility or thinking about broader interactions was necessary. Facilitators used this information as prompts for discussion on Day 2 to encourage participants to add detail to the scenarios. Strategy development activities on Day 2 (see below) were designed to consider the opportunities and challenges in each scenario and in so doing enabled further scenario details to emerge.

### Scenarios As Decision Tools: Strategy Development

The initial draft scenarios were used by participants as decision tools to stimulate small group and plenary discussion within the workshops about strategies which, if implemented in 2016, would result in better, more resilient surveillance systems by 2030 than would otherwise be the case. Participants identified a set of strategies and in a “wind-tunneling exercise” (38), compared the robustness of each across all five scenarios to identify the characteristics of those strategies with broadest application. Subsequently, workshop coordinators performed an in-depth analysis of the perceived strengths and weaknesses of 10 strategies across every scenario to assess the relative robustness of these strategies given the multiple uncertainties present in each future scenario.

### Post-Workshop Activities and Participant Feedback

At the end of the workshop, facilitators were responsible for transcribing each of the scenarios into a coherent narrative, adding more detail to each scenario (using notes of the discussions at each table), and validating the plausibility of the narrative. Participants were invited to comment on the workshop organization and outcomes *via* online and paper-based feedback questionnaires. A draft report summarizing the findings of this workshop and accompanying feedback form was circulated to workshop participants seeking further criticism, feedback, and approval of the final scenarios and strategies proposed.

## Results

Five scenarios were developed. Each scenario incorporates elements of different future consequences from “Brexit,” the UK decision to leave the EU. One scenario explicitly considered the potential consequences of Scottish independence from the rest of the UK, one treated it as largely incidental, one left the political status of Scotland unspecified, and two explicitly stated that Scotland remained part of the UK. Based on a collective understanding of these scenarios, 10 strategies for animal health surveillance were developed and explored and subsequently clustered under three strategic visions.

### Critical Drivers and Scenario Axes

Clusters of high impact and high uncertainty drivers (critical uncertainties) were identified to create three axes (Table 1). The extreme spectrums of each axis were defined by participants and are described below:

1. International trade policy and the importance of the export market: The spectrum of possible outcomes considered ranged from isolationist to globalist policies. Isolationist policies were characterized by an autarkic, strongly Scottish or British focus and less emphasis on multilateral trade agreements. By contrast, globalist policies were seen as promoting open borders, global free trade, and an acceptance of international risk standards for such trade. Brexit and its consequences were included as elements in this axis.
2. Sources for, and availability of, resources for disease surveillance, including expertise and infrastructure: resources for surveillance might be provided by private sources (e.g., by individual companies or industry sectors) or *via* the public sector. In the former case, resources are likely to be more directed to specific industry priorities. In the latter case, government funding, incentives, and priorities may direct surveillance activities.
3. Approaches to data sharing: the spectrum of data-sharing possibilities ranged from highly segregated data management to shared, integrated data resources. Segregated data acquisition and management implies that data are kept in private repositories, and that these data are not shared beyond the entity that collected the data. Furthermore, results from the analysis of these data are likewise not communicated more widely. Data sharing is not required by legislation and is not otherwise encouraged or funded. There is a strong focus on data security and privacy. At the other end of the spectrum, integrated data acquisition and management implies the existence of open, standards-based data platforms and unrestricted data access and sharing (with an opt-out rather than op-in system of participation). Such a system might be underpinned by legislation or funding. Data are likely to be stored centrally and data protection regulations will have evolved further to encourage and allow data sharing. Critically, open data may still be anonymized under specified circumstances.

Other drivers such as farming demographics, environmental impacts on disease distribution, and severity (such as climate change and extreme weather events) were also considered to be important and were included in the discussion and development of each scenario.

### Scenarios

Five scenarios were constructed, each based on a different combination of outcomes from the three axes. Figure 2 illustrates the relationships among these five scenarios, and their positions on the spectra for each of the three critical drivers. Each scenario theme was given a name by participants. Workshop facilitators assigned new names as part of the post-workshop analyses. All names are presented here, so that the text can be cross-referenced to the more detailed stakeholder report (31): Scenario 1. “Free Fall” or “Current Trajectory”; Scenario 2. “Scotland Alone” or “Individual-led Surveillance”; Scenario 3. “Oceania” or “State-led Surveillance”; Scenario 4. “Global Farm” or “Export-led Surveillance”; Scenario 5. “Market Farm” or “Industry-led Surveillance.” Scenarios are described in more detail below and in (31).

#### Scenario 1: The Current Trajectory

This future is characterized by an international trade policy that is neither extremely isolationist nor globalist; a mixed approach to data collection, with some data sharing; and animal health surveillance funded by the state to a moderate degree. This was considered by participants to represent the future, if current trends continue on the same trajectory from 2016, without major shifts in these drivers.

##### Characteristics

In 2030, Scotland remains part of the UK, but after the UK’s departure from the EU, the economy has shrunk. The farming sector has been affected by lower farm gate prices, reduced trade with the EU, and reduced subsidies. There are fewer small professional, family-run farms (i.e., medium or small-scale operations) due to the high volatility of relevant livestock markets and the dominance of larger commercial producers. There are also few newcomers to farming, leading to a generational gap in the farming population and a loss of “institutional memory.” As a result national livestock numbers have declined. Some farms seek to maintain high standards of biosecurity and welfare to protect high-value international trade via new bilateral trade agreements. An increase in trade and travel with non-EU countries, increased movement of people, and climate change have all contributed to an increase in the likelihood of incursion and spread of endemic, emerging, and exotic animal diseases such as liver fluke, West Nile virus, and Bluetongue virus. Some formerly exotic diseases have become endemic in Scotland and the rest of the UK. In this future, surveillance is challenged by limited public resources and expenditure on surveillance, low submission rates to veterinary laboratories and reduced numbers of farmers. Animal health surveillance expertise has been lost, as there are fewer veterinarians in large animal practice in rural areas and less scientific support due to the impacts of post-Brexit immigration policies and a shrinking economy. The disruption of established ties with the veterinary agencies of the EU has made it difficult to access international disease data. Consequently, disease control is now restricted to reactive outbreak management rather than outbreak prevention and early disease detection. Through attrition, some exotic diseases have therefore become endemic in Scotland and the wider UK. There is an increased prevalence of traditional production diseases due to diminished resources and expertise. An exception to the trend is antimicrobial resistance (AMR), the prevalence of which is monitored nationally and there is societal pressure on the agricultural sector to reduce antimicrobial usage. On-farm testing and immediate implementation of control measures allows “drug-bug” coordination (i.e., matching the correct antimicrobial to the correct microbe through rapid and accurate diagnostic testing). There is much uncertainty and variability within and between sectors regarding the prevalence of disease or AMR due to limited data-sharing.

##### Opportunities and Challenges

There is expanding reliance on different technologies (including social media) to obtain and communicate rapid real-time information in outbreaks. Accurate pen-side diagnostic tests and electronic monitoring are available, but this future is characterized by under-utilized technological and data capacity and a shrinkage of surveillance infrastructure associated with a weak economy, fewer human resources, and a lack of data standardization. Smaller farms and veterinary practices will suffer in terms of buying power and social impact. Other farms survive only if they have been quick to invest in, and develop, a niche export market.

#### Scenario 2: Individual-Led Surveillance

This future is characterized by sharply restricted international trade, segregated data management and a lack of public investment in animal health or zoonotic disease surveillance.

##### Characteristics

By 2030, the effects of Brexit and a confluence of other political events have led to Scotland being independent from the rest of the UK and no longer part of the EU. Two main types of farms dominate the agricultural landscape: large industrial or commercial farms and small subsistence farms (which comprise hobby farmers or smallholders, communal farms, and allotments). Small family farms cannot compete with larger, more efficient producers and are declining in number due to the cessation of external subsidies. Profit-oriented, large producers are focused on producing animals with a high health and welfare status. Fewer international trading partners (and less trade overall) reduces competition from cheaper food imports and results in an improved domestic market for produce, but there are fewer foreign workers on farms and higher food prices (due to higher costs and a lack of competition). Livestock and poultry industries are responsible for private funding of animal health surveillance, which companies carry out for their individual benefit. There is reduced value from surveillance data collection, as data are available only within particular companies or at best, industry sectors; data are not shared. There are more “micro”-smallholders and backyard flocks/herds than in 2016, but these are geographically widely dispersed, and have limited funds available to invest in biosecurity or laboratory submissions for surveillance. Consequently, the risk of disease incursion and spread in this sector is high. Illicit animal sales and illegal imports are commonplace. Endemic and even exotic outbreaks (particularly vector-borne diseases) go unnoticed and unreported. The prevalence of AMR is unknown as there are no national surveillance systems in place.

##### Opportunities and Challenges

There is the potential within some large industries and companies to develop technology to increase the speed of disease detection and identification, maintaining high health and welfare status for animals on profitable and competitive commercial farms. Among smallholders, there are opportunities to harness communal resources for disease detection *via* collective community sharing and use of diagnostic assays or other already-available sensor technology, provided it is affordable. Technology is available, but costly, so uptake is limited to inexpensive, robust products that result in significant savings on costs or labor. There is little publicly funded investment in research to support animal health surveillance in Scotland. Limitations on immigration after Brexit have resulted in a “skills-gap” and fewer international researchers are attracted to research opportunities in Scotland. Although there is capacity for data collection, there is limited ability to collate it. What data does exist may be of high-quality and well-targeted to the needs of end-users. The large numbers of new entrant farmers among very smallholders has resulted in a loss of “institutional memory” regarding disease outbreak preparedness.

#### Scenario 3: State-Led Surveillance

This future is characterized by an isolationist trade policy, an integrated state-driven approach to data collection and sharing and state-funded animal health surveillance.

##### Characteristics

In 2030, Scotland is still part of the UK. As a consequence of Brexit, it has lost access to EU markets, resulting in increasingly isolationist policies designed to protect the UK economy. Scotland’s agricultural trade policy is based on a precautionary risk-based approach, which results in fewer, highly selective transactions with trusted partners to reduce the risk of notifiable disease incursion. The UK is no longer a good base for production for external markets; multinational companies have relocated elsewhere. Sheep and beef farms are increasing in size, with more sheep and beef cattle moving to lowland areas, and marginal regions dropping out of production. Remaining pig and poultry units have restructured into smaller units to supply national demand. Due to decreased animal movements, strong import controls and increased biosecurity on farms, there is a decreased likelihood of exotic disease incursion and spread. State-funded animal surveillance programmes increasingly focus on compulsory data collection and sharing on production diseases and endemic diseases, as increases in national production are politically and socially important, and provide clear economic advantages to producers. AMR prevalence is monitored nationally and there remains pressure on the agricultural sector to reduce antimicrobial usage, in keeping with the overall aversion to risk.

##### Opportunities and Challenges

As a result of data-sharing legislation, a large volume of farm health status information is freely and openly available to the public. There are opportunities for new training initiatives focusing on data collection, analysis, and interpretation. It is possible to “benchmark” lower, more sensitive, thresholds for intervention at pre-clinical disease stages, leading to improved early disease detection. The lack of privacy regarding sensitive business and animal health data poses potential economic or business risks to some producers. There is a reduced farm labor force and less veterinary input into farms. It is difficult to attract students (particularly international students) to veterinary schools and agricultural colleges, which results in increased reliance on para-veterinary professions. The agricultural sector is vulnerable to politically driven shifts in policy and governmental resource re-prioritization. There is limited buy-in among industry stakeholders in government-run surveillance due to a perceived lack of control over their own data, and hence their own industry, which results in poor relationships and communication between industry representatives, policy-makers, and scientists. There are also challenges regarding data analysis including the need for computing capacity to deal with high speed, high throughput, high volume data, and a shortage of analytical expertise in the UK. As data are available widely in society and even to international competitors, there are ongoing concerns about the malevolent use of data.

#### Scenario 4: Export-Led Surveillance

This future is characterized by a globalist trade policy, an integrated state-driven approach to data collection and sharing and state-funded (public resourcing) of animal health surveillance.

##### Characteristics

In 2030, Scotland is part of the UK. Post-Brexit, the Scottish livestock industry is buoyant, competitive, and oriented toward the export market, with some niche product production within Scotland. There is a global trade market in high-end Scottish produce whereby the UK has bilateral export agreements with a number of countries. These trade agreements are contingent upon livestock being free from disease and a transparent chain of testing records to support this. The farming sector closely resembles the industry as it is presently with a mix of small crofters and lifestyle farmers, family businesses, and large commercial farmers. Some small family farms find it difficult comply with the new surveillance regulations and in consequence, struggle to continue to operate. Livestock population sizes are slightly smaller than those in 2017. Surveillance for animal health, public health, and wildlife disease is publicly funded by government, which provides grants and tax incentives to a large R&D sector and is supported by innovative technologies. There is vertically and horizontally integrated data sharing between farmers, veterinarians, and stakeholders within and between businesses and sectors. A key limitation of this export-oriented model of animal health surveillance (which is focused on detection of notifiable diseases and AMR) is that endemic non-notifiable diseases spread relatively freely. There are frequent disease outbreak scares, which stem from the importance of the livestock industry to Scotland and the open nature of surveillance data. These regular alarms result in market volatility. Concern about AMR results in heavy regulation of antimicrobial agents, but there is a black market in pharmaceuticals.

##### Opportunities and Challenges

There are commercial opportunities for corporate veterinary practices and scope for further veterinary specialization due to a burgeoning technology market and innovative diagnostics and research sectors, augmented by specialists in biotechnology and data management. These changes have led to quantitative and qualitative improvements in competition between contractors and suppliers in the veterinary surveillance sector, improving overall performance. The misinterpretation or miscommunication of freely available data results in a series of veterinary health scares. Potentially expensive and complicated technical requirements create a squeeze on small farms, which struggle to adopt the technologies and comply with regulations. Limited broadband access is still an issue in remote areas of Scotland. Disease detection for non-notifiable diseases is neglected, which has led to a decrease in production efficiency.

#### Scenario 5: Industry-Led Surveillance

This future is characterized by a globalist trade policy, a segregated approach to data collection and sharing and private or industry-funded animal health surveillance.

##### Characteristics

After Brexit, Scotland remains part of the UK. Effective marketing to promote the Scottish brand is key in developing and promoting trade. International trade, particularly for the export market, is important and drives much of animal health surveillance. Producers who are willing and able to prioritize efficiency and innovation dominate the farming industry. Industry sectors and large vertically integrated retailers form important substructures and act as “silos” for data and information. Large corporate farm-groups and supermarkets have strong lobbying power, and industry structures protect the interests of these companies. Consequently, smaller farms are declining in number. Data are valued commodities and are not shared beyond the designated business or sector unless there is either an economic justification for doing so or a requirement to fulfill statutory disease reporting to maintain global trade-market access. Surveillance is privately funded, conducted in private laboratories, vertically integrated, and aimed at detecting diseases of the greatest importance to the sector. These include production-limiting diseases as well as exotic or notifiable diseases that could affect trade. Certain sectors, such as high genetic-value beef production, become very successful. Others, such as the pig and poultry sectors, which are accustomed to minimal support and able to build on international links, are able to continue, for the most part, unchanged post-Brexit. The government funds a small element of the surveillance budget and operates a much reduced laboratory system to address public health and wildlife threats. There is a decreased likelihood of incursion and spread of exotic diseases due to heavy investments in biosecurity, focused on diseases of trade importance. Emerging diseases can be detected quickly, provided that detection is not dependent on the identification of a pattern across multiple businesses or sectors. Effective biosecurity and control strategies result in decreased prevalence of those endemic diseases that either significantly affect productivity or are of consumer concern. Consumer pressure has resulted in improved, targeted use of antimicrobial agents, but given the segregated nature of surveillance data; it is not possible to obtain a holistic picture of AMR.

##### Opportunities and Challenges

As there are fewer, larger agri-businesses, it may be feasible to obtain data from most, if not all businesses if this can be negotiated between industry partners. However, public health surveillance is not prioritized widely and there is a systematic risk of failure to detect novel diseases due to weak data-sharing. As commercial benefits drive investment in surveillance, there is, at best, limited state access to animal (livestock, companion, and wildlife), human and environmental data to give an overview of the epidemiological situation. There is a loss of farming heritage, skills, and institutional memory about disease, particularly among the reduced number of small farms.

### Strategies to Improve Resilience

None of the proposed scenarios will be an “accurate” description of the future. Their purpose is to facilitate understanding of current trends by exploring possible alternative outcomes. Some scenarios may, however, turn out to be more relevant than others. There are different precursor signs for all five possible futures already present today (Table 2); identification or intensification of such signals might be interpreted as evidence that the associated future should be assigned a higher salience.

**Table 2:** A cross-comparison of scenario characteristics.

	Current trajectory	Individual-led surveillance	State-led surveillance	Export-led surveillance	Industry-led surveillance
Increased tariffs subject to WTO rules?	Yes^a^	Yes	Yes	No	No

Increased imports?	No	No	No	No	Yes
	Decreased	Decreased	Decreased	Decreased	

Increased exports?	No	No	No	Yes	Yes
	Decreased	Decreased	Decreased		

Increased data sharing?	Yes (outbreak response only)	No	Yes	Yes	No (for in-house use only)

Increased value placed on data?	Yes	No	Yes	Yes	Yes

Increased public funding for surveillance?	No	No	Yes	Yes	No
	Industry-led funding increasing	Private individual funding			Industry funding

Reduction in private sector investment in agricultural R&D?	No	Yes	Yes	No	No
	Increased investment in on-farm diagnostics	Reduced demand for investment	Increased public investment in R&D instead		However, reduced public investment in R&D

Increased uptake of technologies to monitor animal health?	Yes	No	Yes	Yes	Yes

Declining numbers of farms?	No	Yes	Yes	Yes	No

Increasing farm herd/flock sizes?	Yes	No	Yes	Yes	Yes

Decrease in veterinary expertise in private practice?	Yes	Yes	Yes	Yes	Yes
			Most vets employed by the state	Most vets are specialists and private contractors	Most vets are specialist industry consultants

Decrease in farmers’ submissions to surveillance centers?	Yes	Yes	No	No	Yes

*^a^This was considered to be the current trajectory in October 2016*.

In each scenario, there are risks that would lead to less effective animal (and public) health surveillance. However, there are also opportunities to improve delivery of animal health surveillance. Workshop participants proposed strategies, which were subsequently examined for resilience (in small group and in plenary exercises) in the context of the five future scenarios. Participants analyzed strategy strengths and weaknesses to explore whether strategies considered desirable and effective in one scenario are irrelevant or even counterproductive under a different set of circumstances. These participant-developed strategies are listed and ranked in Table 3. Subsequently, project investigators clustered these strategies under three strategic visions:

Vision 1. Smart data: strategies to generate and collect surveillance data and improve communication of surveillance intelligence to end-users.
Vision 2. Smart investments: strategies to ensure resilience in human and financial capital resources for surveillance.
Vision 3. Smart users: strategies to address the needs and demands of animal health surveillance end-users and beneficiaries.

**Table 3:** A cross-comparison of participant-developed strategies to improve the resilience of surveillance systems in Scotland in 2030.

	Current trajectory	Individual-led surveillance	State-led surveillance	Export-led surveillance	Industry-led surveillance
**Vision 1. Smart data: strategies to generate and collect surveillance data and improve communication of surveillance intelligence to end-users**					

Industry best	High	High	High	High	Medium
Health risk states scheme	High	Low	Medium	Medium	High
Scottish mobile abattoir scheme	Medium	Very high	Low	Low	Low
Disease intelligence squads	High	Negligible	High	High	Negligible
Surveillance data agency	High	Very low	Medium	Medium	High
Science-policy-industry interface networks for disease exposure and control	High	Very low	High	High	Low

**Vision 2. Smart investments: strategies to ensure resilience in human and financial capital resources for surveillance**					

Rural vet scheme	High	Medium	Low	Medium	Very low
Animal data levy	High	Very low	Low	Low	Medium

**Vision 3: Smart users: strategies to address the needs and demands of animal health surveillance end-users and beneficiaries**					

Digital farming families	High	Low	High	High	Low
Flock-book	Medium	High	Low	High	Very low

*Strategies were ranked by participants according to potential relevance, feasibility of implementation and effectiveness in each future*.

#### Vision 1: Smart Data

Across the posited futures, there is great variation in the nature and efficacy of data collection, and in the ability to analyze and interpret these data, reflecting high uncertainty about future trends affecting these aspects of surveillance. Extrapolating the current trajectory, farmers may invest in technologies for precision agriculture, but there may be fewer farmer submissions and less veterinary resources to generate traditional surveillance data (i.e., clinical samples). If, in the near future, increasingly high volumes of real-time animal, plant, and environmental health data are generated and collected *via* sensors and other emerging technologies, lessons learned from futures such as “State-led surveillance,” “Export-led surveillance,” and “Industry-led surveillance” become more salient. When imagining a future data economy and evaluating the role of data as a commodity, the development of strategic authority over data sharing (including secure data transfer, management, storage, and portability) is as important as technological innovation in smart systems to ensure that data are standardized and integrated to produce information that can be turned into widely accessible and impactful knowledge. In the future, free trade of animal health data, as well as of animal products, may become increasingly important in underpinning Scotland’s economic growth.

Alternatively, if there is little uptake of technology-driven alternatives to traditional data collection activities, other more extreme futures become more plausible (e.g., “Individual-led surveillance”). Both scenarios (high versus low volume and high versus low quality data collection) create the potential for risks and widening inequalities between groups of data “haves” and “have nots.” This, in combination with a non-strategic approach to democratizing (i.e., making publicly available) variable quality information, or significant political shifts toward increased state-control and ownership over data and services, has potential to hasten erosion of public and industry trust in expert opinion and to damage stakeholder perception of the value of investing in a scientific evidence-base for policy. To mitigate these risks, six strategies have been proposed within this vision to address the following:

1.1 Data collection strategies (“Industry Best,” “Health Risk States Scheme,” “Scotland’s Mobile Abattoir Scheme,” “Disease Intelligence Squads”).
1.2 Data-sharing strategies (“Surveillance Data Agency”).
1.3 Communication strategies (“SPIN-DEC”).

##### Data Collection Strategies

Although there are currently “smart” technologies available (in 2016) to collect high volumes of “personal animal data” (such as multi-pathogen screening, biomarkers and data describing animal movement patterns and behavior, etc.), the application and implementation of sensor technology has not been strategic or coordinated within or across sectors. As a result, it is anticipated that future data describing health status, animal behavior, and environmental exposure for individual animals over the long-term, within herds and within farms, could become fragmented and incomplete in certain futures (e.g., “Individual-led surveillance,” “Industry-led surveillance”). Most participants felt that a benchmarking scheme (“Industry Best”) that facilitates data collection and analysis of observations from healthy animals and the environment is an important foundational step. To be sustainable in the long-term, this strategy would require cheap, readily available technology, concurrent investment in telecommunications infrastructure to increase connectivity and expertise for data analysis. Schemes to collect animal health data from a wide variety of sources have been proposed previously (39, 40) but they have not been sustainable over the long-term because of inadequate resources to disseminate the data (40). In the future, a benchmarking strategy could enhance surveillance opportunities in scenarios where data are already freely available and widely shared and analyzed (e.g., “State-led surveillance”) and/or there is available technology to collect on-farm data (e.g., “Current trajectory,” “Export-led surveillance”). However, in futures where there is mandatory data collection and analysis, investment into this strategy may be unnecessary. In an industry-led future (e.g., “Industry-led surveillance”) where the high-quality commercial data is industry-owned, this strategy may not have much traction unless there are sufficient incentives for participation.

There is also future uncertainty about the impact of novel and emerging diseases. These diseases are likely to escape early detection unless farmers actively choose to submit samples, as by definition, there are no mandatory reporting requirements. Participants (in the “Industry-led surveillance future”) suggested introducing legislation for statutory reporting of “health risk states,” i.e., conditions that are not notifiable, but indicate a potentially serious risk to human or animal health to address this knowledge gap. A “Health Risk States Scheme” is a system currently used in human health in Scotland to ensure potential threats to public health are flagged at an early stage based on clinical signs and epidemiology, even if the causative agent is not known (41). This scheme could benefit from co-localization of, and resource sharing between, veterinary and human health laboratories. It could be particularly valuable in futures where data are held by commercial companies, by essentially legislating sharing of early warning signs. Participants thought this strategy would address issues where veterinarians employed by private companies may have conflicts with those companies over reporting early warning signs of potential concern (e.g., in “Industry-led surveillance,” “Current trajectory” scenarios). There is no comparable system in animal health in Scotland, or in international animal disease reporting, where statutory notifications are based on suspicion or confirmation of specific pathogens, although early detection systems such as Programme for Monitoring Emerging Diseases-mail (42) encourage voluntary reporting of similar types of concerning but non-specific information. The strategy would be of limited value in state-run futures where there are already systems in place to manage and analyze data in ways that would encourage early detection of emerging diseases (e.g., “State-led surveillance,” “Export-led surveillance”), or in futures where there is low demand for data and a dearth of relevant expertise (e.g., “Individual-led surveillance”).

There is potential variability and uncertainty about the degree of farmer participation in future surveillance schemes, particularly for small or backyard producers. Submission rates are influenced by trusted relationships between veterinarians, farmers, and the local DSC, as well as disposable income, quality of advice, cost of service, and distance (4). Participants (in the “Individual-led surveillance” future) thought that a “Mobile Abattoir Scheme” could provide a lever to turn traditionally passive surveillance techniques into active surveillance programmes by bringing surveillance to the farmer [see, for example, (43)]. This scheme could deliver on-farm slaughter along-side real-time clinical sampling and robust field-testing to enable rapid detection of endemic and production-limiting disease; information which can be fed back directly to the farmer for his/her benefit. It would also potentially generate data to improve farmer detection of emerging or exotic diseases. To be feasible, this scheme would need to be supported by concurrent investment in technological innovation (pen-side testing), laboratory capacity and data management infrastructure to capture and utilize these data efficiently, as well as education and training for farmers, veterinarians and para-vet technicians. Industry levies or private financing may be important revenue streams for this strategy. Participants suggested that such funding might also come directly from consumers, in the form of a premium paid for the enhanced animal welfare and possible improvements in meat quality that such a scheme might provide. A willingness on the part of consumers to pay such a premium has been identified in at least some situations [e.g., (44)], and a reliance on market forces rather than industry mandates might make such a scheme more palatable to targeted producers. On-farm abattoirs have been used in Sweden (44, 45) and have been introduced to farmers in New Zealand, Australia, and France. However, this is still a niche enterprise and the high costs associated with setup and running costs to ensure compliance with EU regulations may make this strategy unsustainable. If these challenges could be overcome, mobile abattoirs could improve the resilience of clinical data collection in geographically remote and disparate populations of farmers, especially if knowledge about clinical signs is poor and the speed with which diseases will be detected is slow (e.g., “Individual-led surveillance”). It may also be a reasonably useful strategy in futures where there are low rates of sample submissions or where endemic disease is an increasing burden on production efficiency (e.g., “Current trajectory” or “State-led surveillance”). Indirectly, the strategy could also be augmented by implementation of complementary telemedicine (or tele-surveillance) approaches. It is of less value in futures dominated by agri-businesses with high stocking rates, which will require more substantial abattoir facilities to accommodate throughput (e.g., “Industry-led surveillance”). It also lacks relevance in futures with high spending on surveillance infrastructures and point-of-care technologies with mandatory participation and/or high investment in R&D (e.g., “State-led surveillance,” “Export-led surveillance”).

Even if there is an abundance of accessible, high-quality surveillance data in the future, there may be significant challenges in coordinating real-time data analysis and disease control response. Participants (in the “State-led surveillance” future) thought that strategies that create teams of veterinarians, para-veterinarians, technicians, and nurses (i.e., “Disease Intelligence Squads”) who are trained to address this problem *via* interpretation of early warning signals could be beneficial, particularly if the state is posited as both enforcing the collection and sharing of data to promote efficient livestock production. Similar strategies have already been implemented to create a global early warning system (46). “Disease Intelligence Squads” could also be seen as a natural development of the current work of, for example, the UK APHA Pig Expert Group, and their quarterly GB Pig Diseases Emerging Threats reports (47). The feasibility and sustainability of this strategy within Scotland is contingent on the centralized collection and sharing of high-quality longitudinal data on both healthy and diseased animals to identify thresholds for early detection and intervention at preclinical or clinical stages. This strategy was considered more likely to work well in futures where there is support for veterinary services, and “high tech” diagnostic options for on-farm data collection (e.g., “Current trajectory,” “Export-led surveillance”). Participants felt that “Disease Intelligence Squads” would be of very little value in futures where data collection is limited or data are commercially sensitive, disease control is unfeasible or unaffordable or where there are insufficient trained personnel or resources available to support numerous small-holdings (e.g., “Individual-led surveillance”). It would be redundant in futures where similar in-house expertise is already in place and/or data are too commercially sensitive to share (e.g., “Industry-led surveillance,” “Individual-led surveillance”).

##### Data-Sharing Strategy

In some futures, data sharing may be inhibited by industry control and/or non-compliance with open platform initiatives. Participants (in the “Industry-led surveillance” future) proposed the introduction of strategic investments to support the development of non-profit, independent, cross-sector (animal, human, plant, environment) health data “gate keepers” and promote data sharing (i.e., a “Surveillance Data Agency”). A “Surveillance Data Agency” could be designed to decouple surveillance data from cross-compliance, collate, harmonize, and analyze diverse data sources and demonstrate the benefits to farmers (and other end-users) of a multidisciplinary partnership approach to animal health surveillance. This strategy would necessarily need to be underpinned by a coherent long-term data strategy focused on support of epidemiological objectives. Partners from agriculture, environment, wildlife, and water sectors would contribute to support the running of the agency and commit to provide data, thus gaining access to each other’s data. For this strategy to work, technology must already be available and affordable to collect high resolution human, animal, and environmental health data. It would be most effective in futures where access to data is itself an incentive for participation (e.g., futures in which data are segregated, e.g., “Industry-led surveillance,” “Current trajectory”). However, this strategy might have potential to empower stakeholders by offering an alternative approach, particularly salient in futures where government control is strong (e.g., “State-led surveillance”) or if state-directed sources of surveillance data only focus on exotic, notifiable diseases (e.g., “Export-led surveillance”). Its value would be limited if technologies to collect data are not cheap, robust or readily adopted by farmers, if very few data are collected in the first place (e.g., “Individual-led surveillance”) or if the capacity to leverage the collected data is limited. In addition, there could be teething problems if businesses perceive a potential loss in competitive advantage from participation in the scheme and if attention is not paid to improving data practices across the whole of the data cycle.

Schemes that collate existing data sources to enhance surveillance for endemic diseases are already being trialed within individual farming sectors within Scotland. These overcome issues of potential reluctance to share commercial data because they are organized by the industry sectors themselves via assurance schemes, with members willing to share data within a scheme they already trust. However, their coverage is limited to these members.

##### Communication Strategy

Strategies to improve communication and trust between industry, policy-makers, and scientists [e.g., “Science-Policy-Industry interface Networks for Disease Exposure and Control (SPIN-DEC)”] would marshal reliable evidence and empower veterinarians, farmers, agricultural sector, public health stakeholders, retailers, and supermarkets with expertise and intelligence. Participants felt that a “SPIN-DEC” could be a useful innovation in futures where trade in animals, animal-by-products, and food is an important driver for disease freedom or where veterinary services are run and funded by the state, and are particularly vulnerable to re-prioritization (e.g., “State-led surveillance”) and/or futures where an evidence-base is critical to mitigate the risks of animal disease outbreaks and protect the Scottish brand (e.g., “Current trajectory,” “Export-led surveillance”). Participants anticipated that there could be major barriers to implementation. The ready availability of sensitive production data to a public with a variable ability to assimilate the information was thought likely to give rise to a “lowest-common denominator” media environment in which inaccurate or malicious tropes would easily spread. This might be exacerbated by public distrust of both the governmental and commercial elements of the nascent corporate state. In addition, it was thought likely that there would be systemic weaknesses in the ability of government and the agricultural industries to effectively interpret these data sources themselves, and hence their ability to provide useful information and intelligence to production and retail stakeholders or to rebut “fake-news.” Aspects of this strategy can be seen already as present in, for example, the “Data collection-Analysis-Interpretation-Communication” remit of the private company responsible for the national Animal Health Surveillance System in the Netherlands (48). This strategy would be less relevant in futures where there is no need for an evidence-base to underpin policies on trade or animal health and welfare either because trade is limited (e.g., “Individual-led surveillance”) or industry is already an influential lobbyist (e.g., “Industry-led surveillance”).

#### Vision 2: Smart Investments

In some futures, funding and expert capacity for animal health surveillance activities is expected to decline, particularly in rural areas post-Brexit (e.g., “Current trajectory,” “Export-led surveillance,” and “Individual-led surveillance”). Early signals of this may include a reduction in numbers of veterinary school applications, a significant decline in numbers of veterinarians going into livestock practice on graduation, and a reduction in the numbers of veterinarians and veterinary practices in remote, rural areas in Scotland. Public funding cuts for disease surveillance in the face of ongoing or emerging disease threats also increase the salience of these outcomes. To mitigate these resourcing risks, two strategies were proposed:

2.1 Strategy to increase veterinary and scientific research capacity (“Rural Vet Scheme”).
2.2 Strategy to increase surveillance (“Animal Data Levy”).

##### Strategy to Increase Veterinary and Scientific Research Capacity

There is uncertainty about the future availability of human resources and expertise in veterinary services and scientific research, particularly in remote rural areas of Scotland due to a predicted “brain-drain after Brexit” (49) (see for example: “Current trajectory” and to a lesser extent, “Export-led surveillance,” and “Individual-led surveillance”). Participants (in the “Current-trajectory” future) felt that incentivization strategies might be necessary to attract and retain expertise in Scotland and enable better delivery of on-farm testing and data collection to improve endemic disease surveillance and control. Incentivization strategies are commonly used in the medical field to encourage doctors to work in rural areas (50). A large expert opinion study (51) identified debt relief programs as the most supported strategy for increasing the number of food supply veterinarians. A “Rural Vet Scheme” strategy would provide education bursaries or grants to attract and retain veterinarians in large animal practice in rural areas. It would also include incentives for farmers to utilize these veterinarians to ensure that there is adequate demand for the services; participants thought it could be similar to the existing Highlands and Islands Veterinary Services Scheme (HIVSS) that subsidizes veterinary support in remote areas of Scotland providing support to crofters and others of similar economic status (52). In New Zealand, the “Rural Bonding Scheme” is perhaps closer in spirit to the “Rural Vet Scheme” strategy than HIVSS. It goes further than HIVSS and provides support for graduates to ease shortages in rural practices (53). To be feasible in Scotland, this approach requires private or public sources of funding. This strategy would be particularly relevant in futures where there is a dearth of general practitioners – the front-line against disease. However, it is of limited value in futures where the career-path for veterinarians is predominantly within government (e.g., “State-led surveillance”) or in agri-businesses with a strong demand for in-house veterinary services or specialized practices (e.g., “Industry-led surveillance”). Furthermore, the long-term sustainability of the strategy would be doubtful if there was insufficient demand from producers for veterinary services.

##### Strategy to Increase Surveillance Funding

There is also uncertainty about the future availability of surveillance funding and the accessibility of data for industry, as well as government use. Participants (in “Current-trajectory” surveillance future) proposed that new revenue streams be funded through public–private partnerships to encourage industry participation in surveillance and ensure that data are widely accessible. This could include an “Animal data levy” charge for industries, which grants them access to data. The use of a levy is a well-established funding mechanism for agricultural research [(54), at p. 138] and has precedents in the UK such as the levy-funded DairyCo organization, which plays an important role in conducting research and controlling diseases such as Johne’s Disease (55). A levy strategy would require cooperation and collaboration between funders and decision makers. Investors would need to see benefits from their funding and perceive value from access to data. It could return power to the industry (56), mitigate any disconnect between industry and policy, and reduce the impact of any future decline in public funding or reallocation of taxation-derived resources away from surveillance. Tabor et al. (54) (at p. 140) suggest that globalization and liberal trade policies erode the “public good” aspect of agricultural research and other policies, causing non-public funding mechanisms to become more important. Following this argument implies that a levy strategy would be more relevant in non-isolationist futures (e.g., “Current trajectory,” “Industry-led surveillance”), but would be of limited value where the sector is not economically viable or there is no industry solidarity (e.g., “Individual-led surveillance”). It may lack relevance in futures where data are already publicly funded and freely shared (e.g., “State-led surveillance,” “Export-led surveillance”). Public–private partnerships are likely to contribute to “One Health” approaches to healthcare and contingency planning and would be feasible and effective if implemented. However, if companies are forced to share all of their data as a condition of access, there may be some resistance to uptake in such futures (e.g., “Industry-led surveillance”). Furthermore, the strategy might be unsustainable in futures with little emphasis on export or imports, and hence less demand for strong surveillance frameworks.

#### Vision 3: Smart Users

In most proposed futures, technology is an important driver for development and improvement of animal health surveillance (e.g., “State-led surveillance,” “Current trajectory,” “Export-led surveillance,” and “Industry-led surveillance”). These futures would be evidenced by increased volumes of “Big Data” routinely collected from growing numbers of competitive farm businesses. It is also anticipated across most futures that shifting demographics of farming in Scotland and the UK (i.e., toward new agri-business entrants and small-holders and away from traditional family farms) and/or a drop in research investment (which would reduce data analytic support) would result in more demand that end-users (i.e., clinicians, farmers, livestock keepers, and agricultural workers) be able to critically analyze such data if they are to derive the available benefits. This may create further pressure on lifestyle farmers and a resultant loss of certain aspects of Scottish farming heritage. To mitigate these challenges, strategies have been proposed to:

3.1 Improve digital literacy of farmers so they (and their successors) can participate in the data economy (“Digital Farming Families”).
3.2 Improve industry solidarity and disease expertise (“Flock-book”).

##### Strategy to Improve Digital Literacy

In the future, there may be important skills gaps in agricultural data analysis, digital literacy in farming data informatics (for all ages) and technological expertise. Participants (e.g., in the “Export-led surveillance” future) proposed a targeted, grant-funded data-skills training scheme for farming families in rural Scotland (“Digital Farming Families”) to provide digital literacy education at all levels, with a specific application of such skills to farming needs. This would enable successive generations of farmers to be prepared for technological changes as they occur and enable farmers to access relevant surveillance outputs and make use of these resources themselves. This would be contingent on R&D funding and research innovation to ensure there are technologies available for precision agriculture. The demand for precision agriculture and disease detection may also depend on international standards for trade risks and on non-tariff barriers to trade. The strategy would be most relevant in futures where there is a heterogeneous landscape of farming types (from crofters, to lifestyle farmers and family businesses as well as large-scale agri-businesses) and there are clear farming legacies and succession planning for the next generation of farmers. There would be obvious benefits in any future where there is a knowledge gap between technology and end-users (particularly if implementation of technology is mandatory), a lack of buy-in to any informatics-oriented strategy from the farming community and a need for skilled expertise (e.g., “Export-led surveillance,” “State-led surveillance,” “Current trajectory”). It would be of less value in futures where there is a low demand for technology (either due to lack of availability, affordability, or perceived benefits) (e.g., “Individual-led surveillance”). It may be less relevant in futures that are dominated by large agri-businesses, which already have access to this training and expertise and are expressly not the target market (e.g., “Industry-led surveillance”). In this regard, it serves to prevent the burden of mandatory changes in data recording from falling disproportionately on smaller businesses. It has parallels in a number of government schemes aimed at small and medium sized enterprises (SMEs) and initiatives to assist smallholder farmers.

##### Strategy to Improve Industry Solidarity and Disease Expertise

In some of the posited futures, it is expected that there may be further reductions in the number of farmers (and traditional farming families) who have experience and knowledge of previous outbreaks (e.g., Foot and Mouth Disease in 2001). Participants (in the “Export-led surveillance future”) thought that a social media platform would be of particular use to smaller farm businesses to address a gap in knowledge, communication, and real-time data analysis (“Flock-Book”). “Flock-book” is targeted at farmers to facilitate transparent data sharing, communication, and analysis of animal surveillance data (particularly for non-notifiable diseases). The platform would be underpinned by algorithms that process and analyze data in real-time. The system would be farmer-owned and led, on a mutual basis. There could be opportunities for this to be a commercial business, generating income for members through online advertising. It would necessarily be underpinned by R&D investment to develop new technologies and data analytics and would require broadband connectivity to work. It would be relevant in futures where farmers need to empower themselves (for example, in the face of strong state regulation and social-media informatics-driven criticism), improve sector solidarity, or find new opportunities for early warning systems and ways to reduce time-to-detection (e.g., “Individual-led surveillance,” “Current trajectory,” “Export-led surveillance”). It could be particularly relevant in futures with strong social-media information-driven criticism of industry sectors. However, it could be difficult to implement if there was little demand for and/or few adopters of the platform. Participants felt the success of the strategy would be heavily reliant on active participation by all relevant stakeholders. If a small group of stakeholders does not subscribe or subscribes but does not contribute, this might undermine both the quality of and stakeholder confidence in the data system. The strategy would be redundant in futures where demand for infrastructure and training was already met by market forces (e.g., “Industry-led surveillance”) or government (e.g., “State-led surveillance”).

## Discussion

The EPIC scenario planning workshop produced five diverse and plausible views of the future of Scottish animal health surveillance. These scenarios highlight a number of important and influential drivers that have the capacity to affect long-term resilience of early disease detection and control of exotic, endemic, and novel animal and zoonotic diseases. The scenarios were broadly defined by three axes: international trade policy, data management and data-sharing philosophies, and sources of finance for surveillance infrastructure and capacity. The process of creating these scenarios required consideration of what livestock industries might look like in a future Scotland, including factors such as farming structure and demographics, farming education, and technology uptake.

The scenarios also enabled participants to think about creative strategies to mitigate risks and maximize opportunities to improve surveillance. In the absence of any certainties about the nature of post-Brexit trade agreements for agriculture, the most robust strategies (i.e., those thought likely to be effective, feasible, and relevant in most futures) and thus the best investments for long-term resilience of surveillance systems included data collection strategies (i.e., “Industry Best” and “Health Risk States Scheme”), user-benefit strategies (“Digital Farming Families” to improve digital literacy in farming communities), and investment strategies to increase veterinary and scientific research capacity (“Rural Vet Scheme”) (Table 3). These strategies highlighted three areas for further strategic consideration: “smart systems” (Vision 1), “smart investments” (Vision 2), and “smart users” (Vision 3) to ensure there is a market (and therefore a mechanism to generate resources) for new surveillance systems. Some of these strategies represent novel approaches, while others have aspects that are currently in use or being trialed in Scotland or other countries. This scenario planning exercise has illustrated how these approaches might be developed further to address particular threats or opportunities. Given that there were some parallels or overlaps with existing systems in Scotland and elsewhere, it is possible discussions may have been overly influenced or dominated by participants already working in veterinary surveillance. However, the inclusion of strategies based on other fields, for example, the health risk states scheme from human medicine, illustrates the value of including a broad participant expertise base.

### Future Farms

Consideration of industry structure was a prerequisite for subsequent exploration of the requirements, structure, and limitations of surveillance in each scenario. Future resilience planning for key Scottish livestock industries (i.e., sheep and cattle) has been addressed in detail by previous foresighting work (30, 57, 58). It is not known whether participants in the current exercise had accessed these reports prior to the workshop. However, across all these workshops, participants appear to have held consistent views regarding the importance placed on drivers such as market access (exports and imports), government support (for farms and/or for surveillance), and technological innovation. This is evidenced by the fact that in both this and the previous scenario planning workshops (30, 31), participants envisioned a similar group of plausible, but diverse futures for farming.

Any one of the five futures proposed in this workshop is possible (Table 2). However, the hypothesized future timelines indicate that there are likely to be periods of significant divergence during which the hypothetical trajectories leading to these different futures would take radically different directions. Important signals to monitor for divergence would include trends in farmer demographics, technology uptake, attitudes toward data commoditization, surveillance submission rates (by current mechanisms), significant political shifts and changes in public perceptions of evidence. However, the most important influence on the positioning of the “real” future relative to the five posited futures is likely to be the nature of post-Brexit trade agreements applying to agricultural produce.

### Trade

At the initial time of writing, the official stance of the UK government was that it would not seek to remain a full member of the EU customs union, so that the UK would have freedom to negotiate comprehensive trade agreements with non-EU countries (59). This position is now more uncertain after the June 2017 UK General Election. However, if this is the future course for the UK, it may push Scotland nearer to “Individual-led surveillance” or “State-led surveillance” futures (in which WTO tariffs apply), unless preferential free trade agreements (which include agricultural products and services) can also be negotiated with the EU (or an independent Scotland rejoins the EU as a new member state). In the absence of a UK–EU agreement, there may still be beneficial impacts on farmgate prices for some sectors (e.g., cattle) as EU imports are unlikely to be competitive. However, new risks from low-cost international producers may emerge depending on whether the UK retains the EU’s non-tariff barriers (i.e., the ban on beef treated with growth hormones) (60). Other sectors (e.g., sheep meat) will be at greater risk if tariff-free access to the EU market is not secured (60, 61). Any gains or losses due to transaction costs would also need to be counterbalanced against the loss of Common Agricultural Policy support and reduced availability of public funds to spend on animal health and surveillance activities. Changes in farm income will necessarily impact on whether farmers are able to continue farming, invest in technology, pay for veterinary services, and access and contribute to the cost of disease surveillance schemes. Trade policy (and choice of trading partners) also affects the fundamental purpose and objectives of surveillance activity. Futures that depend on an export market (e.g., “Industry-led surveillance,” “Export-led surveillance”), need surveillance systems which are focused on diseases important to trade but this prioritization may leave gaps in surveillance in other important areas (such as production and endemic diseases, wildlife, and public health).

Despite the uncertainty over trade policy, the results from the workshop suggest that a strong “Scottish brand” should be encouraged and promoted by industry. Sustaining this brand will depend on industry self-sufficiency, solidarity and coherent messaging (all of which are contingent on improved ICT, data management/sharing and delivery of veterinary surveillance services, particularly in remote areas in Scotland). These investments were identified as necessary in ensuring that future farm demographic changes do not result in a loss of disease management expertise and lower disease vigilance. Such a risk would manifest if there is a shift toward more efficient large-scale commercial businesses and/or very small-scale, backyard farming or a polarized situation including both. In every future developed in this workshop, participants considered that the lifestyle or family farmer might disappear completely, raising important questions about succession planning and the value placed on the family-farm as part of the structure of Scottish rural society.

### Resources for Surveillance

In every future, the source of funding influenced, in broad terms, the anticipated design and implementation of surveillance systems. This confirms the importance of thinking about surveillance, not only as an epidemiological activity but also as an economic one (5). Scenarios in which surveillance was government-funded saw more efficient cross-sector monitoring and control of important hazards like AMR. Futures in which surveillance was industry-led and funded exhibited advantages from better surveillance within vertically integrated systems “from farm to fork,” and from organized sectors being able to prioritize control of diseases important to the industry. However, the latter left potential gaps in wildlife, public health, emerging disease and potentially endemic disease surveillance, raising questions over where a limited government budget would best be deployed. Several of the proposed strategies, such as public–private partnerships or incentivized data-sharing schemes, were aiming to mitigate concerns that industry-led surveillance might not promote data sharing or public health. The proposed “Surveillance Data Agency” also recognized the need to attach a proprietary value to data, allowing data (and knowledge) to be exchanged freely, but exclusively, within a “well-defined network of relationships” (62).

### Data-Sharing Philosophies

The emphasis on data (and specifically, data-sharing philosophies) rather than technological innovation (57, 58) as one of the three scenario axes may have had interesting implications for the way in which stakeholders perceived the future. In particular, the nature of data control and ownership may influence the perceived desirability of different scenarios as a function of the social context. For example, government-led futures in which there is a great deal of financial support for farmers and for services such as surveillance may be considered positively by stakeholders if they can be rewarded with greater data control (by exclusion of competitors). However, in futures where government pays for and controls the data (i.e., “State-led surveillance”), there is no foreseeable competitive advantage from data generation and hence this scenario may be considered less favorably (particularly if there is a perception that government could use the data to penalize farmers for failure to comply with regulations). Holistic surveillance was seen as challenging in futures in which data were a commodity shared only within commercial companies. The impact of this logic within the scenarios is reflected in how many of the strategies aimed to either prevent this situation emerging, *via* strategies to demonstrate the up-front benefits of data sharing, or to mitigate the effects of closed data policies, by, for example, incentivizing or legislating for sharing of information. Although scenarios with highly integrated data systems had advantages for surveillance, these were felt to exhibit a potential for false alarms associated with data misuse, exacerbated by the roles of social media and public opinion. This finding highlights a need further to explore stakeholder beliefs and values and is the focus of research to be conducted this year.

### “Brexit”

Although the decision to leave the EU had been confirmed as government policy at the time of the workshop, there was considerable uncertainty regarding the UK negotiation stance, let alone the nature of any final Brexit deal. This uncertainty was compounded by the apparent failure of UK policy-makers to plan in advance for a “Leave” outcome in the referendum (22). This uncertainty was reflected in the workshop discussions. If, in the near future, there is no deal made with the EU, Scotland as part of the UK will be subject to increased tariffs under WTO rules. This would be likely to increase the relevance of certain futures (e.g., “Individual-led surveillance” and “State-led surveillance”) compared to others (e.g., “Export-led surveillance”).

Brexit was considered by workshop participants to be a critical driver for surveillance, with potential to have important but negative impacts on agriculture and animal health research. One participant noted:

Brexit is the biggest life changer for the farming industry since the Second World War… the effect of resource cuts both financial and personnel (mean) Brexit has the potential to increase the animal health risk to the whole of Great Britain.

Participants anticipated that it may become difficult to attract and keep researchers and operational staff with animal and zoonotic health surveillance expertise to work in Scotland and the UK, and farmers may be less able to pay for clinical and pathology services. Although the consequences of Brexit for farmers are highly unpredictable, it is difficult to believe that they will be advantageous [(63), at p. 11] because of the potential for the removal of direct payments, reduced market access and competition from cheaper imports. Other implications of Brexit were also discussed, including changes to pharmaceutical regulatory structures, which may in turn influence R&D investment, access to other types of research expertise, medicines, and new diagnostics.

The identification of Brexit as a critical driver in this study may be usefully contrasted with discussions in previous scenario planning workshops (57, 58), where the, then pending, referendum on Scottish Independence was not selected for discussion in detail as it was neither considered to be highly important nor uncertain. Independence was seen as having little impact on the evolution of the sector because of assumptions about epidemiological and political constraints (i.e., that the UK would remain a single epidemiological unit, that the budget for Animal Health was already devolved to Scottish Government, and that an independent Scotland and the residual-UK would both ultimately trade within the European Single Market under common regulations). Elements of continuity post-independence were seen as more important than those associated with political change. This is not true of the changes arising from Brexit.

The effect of Brexit as an unexpected “shock” event dominated aspects of scenario development and as a result, perhaps for some participants, limited deeper discussion of genuinely impactful, but less immediately salient drivers, including those whose own uncertainty have been radically increased by Brexit. However, had the workshop been held prior to the referendum vote, it is by no means certain that a Brexit-type event would have been included as a critical driver. Some of the scenarios arising from such a workshop might have been informative in navigating the uncertainty arising from a subsequent Brexit decision, but in general it seems likely that many of the outputs would have rapidly become redundant in the light of a Brexit decision. The key operational decision was, therefore, whether to facilitate the inclusion of Brexit as a driver, given that the workshop participants clearly saw it as important and uncertain. As discussed earlier, methodologically, the inclusion of Brexit as an explicit component of the trade critical driver may have been problematic, but it appears to have been the appropriate decision. During the period over which this paper has been written, the authors perceive the relative salience of the different scenarios as having changed in response to different political events and pressures. However, we believe that at all times, at least some of the scenarios can be seen as having relevance to the then current situation. This evidences the robustness of the scenario planning methodology during periods of rapid change and high uncertainty.

### Limitations

The original intention of this workshop was to include consideration of disease surveillance in equidae, wildlife, companion animals and people. However, these sectors did not feature strongly in any future described. This may reflect the fact that the dynamics of surveillance in these sectors are substantially different to those in the livestock or poultry sectors. The primary focus on cattle and sheep may reflect the degree of integration within these sectors, compared to the equine industry that has a number of different silos with different priorities. Drivers of change in the racing industry may be very different to drivers impacting on riding schools, owners of companion animals, or the traveler community. Alternatively, the outcomes may reflect the balance in background and interests of the workshop participants, all refracted through the prism of small group dynamics (although representatives from these sectors were invited, did attend, and we believe did add value to the discussions even where these were focused on issues distinct to their sectorial experience). The opinion of the authors is that, where the lessons learned from this study are not easily transferrable to other sectors, there would be value in holding a further workshop to identify sector-specific issues associated with the future of surveillance.

Participant diversity, the time available for discussion, and the particularity of contextualized data elicited from discursive approaches are recognized limitations of a scenario planning approach (17, 64). Workshop dynamics were not explicitly evaluated as part of this study. Our subjective assessment (supported by participant feedback) was that improvements in the room layout, the time allocated for discussion, smaller group sizes, and more effective facilitation of some of the group exercises could have influenced and improved group dynamics. Nevertheless, judging from this feedback, the participatory process was also a success; participants felt “the evolution of the process was novel and thought provoking,” created new relationships and were challenged to think creatively “outside the box” by different multidisciplinary viewpoints.

## Conclusion

Against a background of increasing population growth, climate change, and political uncertainty, future animal health surveillance activities must support better animal health and productivity to ensure global food security and safety. These drivers are not unique to Scotland, and as such, the strategic visions (“smart data,” “smart investments,” “smart users”) identified in this workshop are likely to be relevant to other, similar, developed countries. In a UK context, the strategies identified in the workshop (such as “Industry Best,” “Health Risk States Scheme,” “Rural Vet Scheme,” and “Digital Farming Families”) as the most robust (i.e., relevant, feasible and effective) should be explored and considered further by industry and government stakeholders as opportunities to improve the long-term resilience of surveillance beyond Brexit.

Future challenges for surveillance are undoubtedly complex and often “incalculable” (65). Scenario planning offers a structured, robust approach to “render futures actionable, when the future cannot be known” (65). It enables consideration of non-probabilistic “what-if” scenarios rather than considering desirable or probable futures and offers an opportunity for constructive dialog at the interface between science, society, and policy. This reflexive approach is not just about improving anticipatory governance but rather, emphasizing the promotion of parallel partnerships between governance and society in the face of uncertainty to improve the future (30, 66). In the Scottish context, stakeholder “ownership” of animal health surveillance is perceived to be vital to promoting acceptance of any changes made to future delivery systems (4). We believe the discussions and relationships between participants in government, industry, and academia during this process (and the challenges this brought to established thinking about veterinary surveillance) are what make this approach to surveillance planning, novel and particularly important light of the uncertainties associated with Brexit. As such, we hope that this scenario planning workshop will have a positive impact at both the policy level where stakeholder buy-in and input are advantageous, and at the industry level where innovation and good practice will be encouraged. Offering opportunities for this type of dialog, to explore differences in values and interests and to resolve potential conflicts between stakeholders, is likely to become even more important as the UK takes steps to negotiate a Brexit deal. UK policy-makers may have an opportunity to design “new food, farm and environmental policies, best suited to British circumstances” (22) but surveillance will have huge importance in this context, as they will also be expected to protect the high standards of animal health and welfare in Scotland, protecting the interests of both Scottish farmers and consumers at the same time as responding to other global challenges.

## Ethics Statement

This study was carried out in accordance with the recommendations of University of Glasgow College of Medical, Veterinary and Life Sciences Ethics Committee for Non-Clinical Research Involving Human Subjects with informed consent from all subjects in accordance with the Declaration of Helsinki. The protocol was approved by the University of Glasgow College of Medical, Veterinary and Life Sciences Ethics Committee for Non-Clinical Research Involving Human Subjects.

## Author Contributions

LB was responsible for the concept, design, and writing of the manuscript and led the scenario planning workshop activities. LB, HA, AR, PB, GR, and IM were responsible for workshop facilitation and data collection. HA, AR, PB, GR, and IM made contributions to the content and critical revision of the manuscript. All authors are accountable for the accuracy and integrity of this work and have approved the final manuscript for publication.

## Conflict of Interest Statement

None of the authors of this paper have a financial or personal relationship with other people or organizations that could inappropriately influence or bias the content of the paper.

## Supplementary Material

Scenario planning: drivers of change. Available at: http://www.epicscotland.org/our-research/encouraging-interdisciplinarity/scenario-planning/scenario-planning-the-future-of-animal-health-surveillance-in-scotland/drivers-of-change/.
